# Autosomal STR Markers for Forensic Genetics: Applications, Challenges, and Future Directions

**DOI:** 10.3390/genes17030285

**Published:** 2026-02-27

**Authors:** Irena Zupanič Pajnič

**Affiliations:** Institute of Forensic Medicine, Faculty of Medicine, University of Ljubljana, Korytkova 2, 1000 Ljubljana, Slovenia; irena.zupanic@mf.uni-lj.si; Tel.: +386-1-543-72-15

**Keywords:** autosomal STRs, forensic genetics, massively parallel sequencing, human identification

## Abstract

Autosomal short tandem repeat (STR) markers remain the cornerstone of modern forensic genetics, providing exceptional power for individualization, kinship verification, and reconstruction of complex investigative cases. Over the last decade, the field has undergone a major technological transition from length-based capillary electrophoresis (CE) toward sequence-level characterization using massively parallel sequencing (MPS), enabling detection of internal sequence variants (isoalleles) and flanking-region polymorphisms that substantially increase discriminatory power in many forensic contexts. Although MPS is increasingly adopted in forensic laboratories, implementation remains dependent on infrastructure, cost considerations, validation requirements, and jurisdiction-specific legal frameworks. This review synthesizes the molecular mechanisms underlying STR variability, including replication slippage and mutation processes, and critically evaluates the transition to sequencing-based analysis. Particular attention is given to analytical challenges such as stochastic effects in ultra-low-template DNA and PCR inhibition in degraded samples. Special emphasis is placed on identification of skeletal remains from mass graves and historical contexts, where hierarchical analytical strategies—from mini-STR approaches to MPS-based workflows—enable recovery of highly fragmented DNA. The review also examines the evolution of probabilistic genotyping (PG), highlighting the importance of algorithmic transparency and reproducible analytical frameworks for judicial applications. By integrating technological advances with practical forensic challenges, this review outlines a comprehensive framework for implementing high-resolution STR analysis in contemporary genomic casework. As a narrative synthesis, the conclusions reflect currently available published evidence and acknowledge variability in validation status, implementation practices, and regional forensic infrastructures.

## 1. Introduction

DNA typing currently represents the most robust and authoritative methodology for individual identification and kinship verification within forensic medicine and criminal investigations [1,2,3,4]. To establish highly discriminatory genetic profiles, forensic practitioners utilize short tandem repeats (STRs)—hypervariable autosomal loci characterized by tandemly repeated nucleotide motifs distributed throughout the human genome [5,6,7]. These markers are considered neutral genetic elements, as they reside primarily in non-coding regions and do not convey phenotypic or health-related information, thus adhering to the stringent ethical and legal requirements of identity-only profiling [1,6,8].

Historically, forensic individualization and paternity testing were restricted to the analysis of protein polymorphisms, targeting erythrocyte antigens, serum proteins, and enzymes [9]. These biochemical markers, however, exhibited limited heterozygosity and low power of discrimination, often proving inadequate for definitive identification or complex kinship confirmation [10,11]. Such methodologies were largely exclusionary and significantly hampered by the rapid environmental degradation of proteins and the minute quantities of biological material typically recovered from crime scenes [2,12].

The emergence of molecular genetics facilitated a paradigm shift toward the direct analysis of hypervariable DNA regions. DNA polymorphisms offer superior advantages, including high environmental stability and an abundance of variable sites throughout the genome [4,13,14]. This evolution was accelerated by the development of the polymerase chain reaction (PCR), which allowed for the amplification of low-copy-number DNA from trace evidence, such as hairs, bones, or touch DNA samples [1,6].

In contemporary forensic practice, the field is undergoing a second major transition: from length-based analysis via capillary electrophoresis (CE) to sequence-based characterization using massively parallel sequencing (MPS), also known as next-generation sequencing (NGS) [3,11]. This transition addresses the inherent limitations of CE, particularly in resolving complex biological mixtures and analyzing compromised samples. Furthermore, the strategic integration of autosomal STRs with supplementary markers is increasingly utilized to address the challenges posed by highly degraded biological material, such as that encountered in historical investigations and mass disaster victim identification [15,16]. This review aims to examine the molecular characteristics, mutational dynamics, and analytical advancements of STR markers, providing a comprehensive overview of their critical role in modern forensic identification and the emerging methodologies that define the future of the field.

## 2. Methods of Literature Review

This study was conducted as a structured narrative review aimed at synthesizing current knowledge on autosomal STR markers and emerging sequencing-based forensic methodologies. Literature searches were performed using PubMed, Web of Science, and Scopus databases employing combinations of the keywords “autosomal STR”, “forensic genetics”, “massively parallel sequencing”, “probabilistic genotyping”, and “degraded DNA.” Publications between 2000 and 2024 were prioritized, together with foundational earlier studies relevant to methodological development.

Eligible publications included validation studies, population genetic analyses, international guideline documents, methodological evaluations, and applied forensic casework reports. Studies lacking methodological transparency or forensic applicability were excluded. Particular emphasis was placed on peer-reviewed publications and recommendations issued by the DNA Commission of the International Society for Forensic Genetics (ISFG) [2,3,7].

Because this review follows a narrative rather than a systematic design, a formal meta-analysis was not performed. Instead, evidence was interpreted qualitatively, considering methodological robustness, reproducibility, and practical forensic applicability.

The methodological strength and potential bias of included sources were qualitatively assessed according to study type and evidentiary contribution (see Supplementary Material, Table S1).

## 3. Genomic Organization and Molecular Characteristics of STRs

STR loci are highly polymorphic regions of the human genome that serve as the primary markers for genetic individualization and kinship analysis [9,17]. Predominantly located within non-coding segments, such as introns and intergenic sequences, STRs constitute approximately 3% of the total human genome [6,9]. Although historically categorized as non-functional DNA, contemporary research indicates that these loci play vital roles in transcriptional regulation, gene expression modulation, and the maintenance of chromatin structure [17,18,19]. Forensic STRs are specifically selected for their high heterozygosity and independent Mendelian segregation, which provides the theoretical foundation for robust statistical interpretations [6,20,21]. While core motifs range from two to seven base pairs (bp), tetranucleotide repeats remain the forensic standard due to their reduced propensity for stutter artifacts and superior allele resolution [3,22,23].

The analytical paradigm has recently shifted from traditional length-based polymorphism to sequence-level characterization facilitated by MPS. This technology has revealed that STR variability is significantly more complex than previously recognized, uncovering hidden variation known as isoalleles—alleles that are identical in length but distinct in their internal nucleotide sequence [24,25]. Such sequence-level information includes single-nucleotide polymorphisms (SNPs) within the repeat motifs, flanking region variations, and complex compound structures [14,24]. The integration of this high-resolution data substantially increases the power of discrimination, which is particularly critical for the deconvolution of DNA mixtures and the analysis of highly degraded samples [14,25]. Furthermore, sequence-based analysis provides deeper insights into locus-specific mutation patterns and the molecular determinants of stutter formation, ensuring more accurate population statistics and refined forensic interpretations [8,26,27].

## 4. Molecular Mechanisms of STR Variability

**Figure 1:**
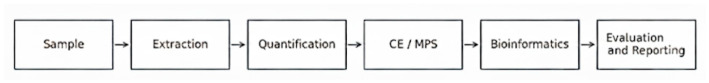
Conceptual forensic genetics workflow integrating sample collection, DNA extraction, quantification, STR amplification using CE or MPS platforms, bioinformatic processing, and statistical interpretation. Performance at each stage is context-dependent and influenced by sample quality, laboratory validation, and analytical thresholds.

The overall analytical workflow discussed across Section 4, Section 5, Section 6, Section 7, Section 8, Section 9, Section 10 and Section 11 is summarized in Figure 1, illustrating the relationship between sample processing, STR analysis technologies, and downstream statistical interpretation.

STR loci exhibit continuous expansion and contraction, accounting for their substantial allelic length variation among individuals [17,20]. This polymorphism primarily results from mutational events, such as insertions and deletions, which alter the tandem repeat count and subsequently modify the locus length [4,12]. The principal mechanism driving STR instability is replication slippage—or slipped-strand mispairing—during DNA duplication [12,26]. The formation of secondary DNA structures, including hairpins and loops, can induce the DNA polymerase to slip at the replication fork, thereby generating novel alleles [26,28].

Replication slippage can lead to either a decrease or an increase in the number of repeats. A reduction occurs when single-stranded loops form on the template strand; the DNA polymerase bypasses these secondary structures, resulting in a deletion. Conversely, an increase in repeat number is caused by the formation of hairpin-like structures on the nascent DNA strand, which are subsequently duplicated in the following replication cycles, leading to an insertion [29,30]. This slippage also occurs during PCR, generating by-products known as stutter artifacts. While these artifacts complicate the analysis of dinucleotide and trinucleotide STRs, tetranucleotide repeats produce significantly lower stutter ratios, reinforcing their forensic utility [3,4,22]. Recent MPS studies have clarified that stutter formation is determined by sequence-level factors, including repeat number, flanking sequence composition, and total allele length [17].

The mutation rate of STRs is further influenced by sequence purity, motif length, and the presence of interruptions. Perfect repeats are highly prone to expansion or contraction, whereas interrupted repeats exhibit significantly lower mutation rates and greater structural stability [4]. Additionally, STR instability is modulated by the DNA repair machinery, particularly mismatch repair systems; deficiencies in these pathways increase the frequency of slippage-induced mutations [11]. Population genomic studies indicate that these mutation patterns vary across different genetic backgrounds, reflecting distinct selective constraints. Such insights are indispensable for forensic applications, specifically for calculating match probabilities or evaluating kinship, where locus-specific mutation rates directly affect the probability of observing allelic discrepancies [6]. High-resolution sequencing now enables the direct detection of rare sequence variants and compound alleles, allowing for precise discrimination between closely related individuals and the resolution of complex biological mixtures [4,26].

The molecular mechanisms described above directly influence amplification variability and interpretation uncertainty, which are addressed through stochastic modeling and validation strategies discussed in the following sections.

## 5. Evolutionary and Population Dynamics of STR Mutations

Comparative studies of human STRs and homologous loci in other primates have consistently demonstrated that human STRs exhibit significantly higher mutation rates than those of closely related species [31,32,33,34]. This divergence is largely attributed to the generally longer repeat lengths and higher motif purity found in the human genome, which predispose these loci to replication slippage and multistep mutational events. Evolutionary analyses suggest that human STR evolution is primarily driven by the progressive accumulation of repeat units, with insertions occurring with greater frequency than deletions [5,35]. Empirical studies indicate that approximately 70–75% of mutational events result in an increase in repeat number, supporting a net directional elongation of these loci over successive generations [36].

STR mutations typically adhere to a stepwise model, producing alleles that differ by one or two repeat units, with longer alleles being notably more mutation-prone [5,26,37,38]. While the majority of these events involve a single repeat unit, multistep changes—sometimes perceived as single-step transitions—are rarer but contribute significantly to overall allelic diversity [26,30,39]. Allele length serves as a critical determinant of the mutation rate, as longer alleles mutate more frequently than their shorter counterparts, resulting in a positively skewed distribution of allele frequencies within populations [3]. Germline mutations can lead to apparent exclusions in true kinship cases, as demonstrated by observed single-step mutations at the D13S317 and DXS10148 loci across three generations [35]. Conversely, somatic mutations may cause tissue-specific genotypic differences, which can complicate individual identity testing [5,40]. Overall, STR loci exhibit relatively high mutation rates, ranging from 10^−5^ to 10^−3^ per locus per generation, which far exceeds the genome-wide average for point mutations [20,41,42]. Intrinsic sequence features also heavily influence STR mutation dynamics, and pure repeats exhibit higher mutation rates [4]. The broader genomic context—including flanking sequence composition, chromatin structure, and proximity to recombination hotspots—further modulates the frequency of these events [8,11]. Furthermore, environmental factors such as oxidative stress and specific defects in the DNA repair machinery can exacerbate mutation rates within germline cells [35].

In forensic practice, precise knowledge of STR mutation rates is critical for the accurate interpretation of kinship testing and the statistical assessment of DNA evidence [32,41]. Loci with mutation rates exceeding 1% per generation are generally excluded from close kinship analyses to minimize the risk of false exclusions [4,43]. Empirical studies across diverse populations, involving autosomal, X-chromosomal, and Y-chromosomal STRs, have revealed significant locus-, chromosome-, and population-specific mutational variability [42].

While most STR mutations occur in germline cells, somatic mutations, though less frequent, can complicate forensic analysis if different tissues from the same individual exhibit genotype discrepancies [38,40]. High somatic mutation frequencies are often observed in tumors or cells under replicative stress, emphasizing the necessity of selecting appropriate reference tissues in both forensic and clinical contexts [5]. In paternity testing, germline mutations may produce apparent exclusions that must be interpreted using established guidelines, such as the “triple exclusion rule”, to avoid misclassification [3,26].

Advances in MPS technology now enable the high-resolution detection of sequence variants, compound alleles, and sequence interruptions within STR loci [24]. These analytical approaches provide profound insights into mutational mechanisms that remain undetectable by traditional CE, thereby improving the accuracy of population-specific allele frequency databases [24,26]. Accurate mutation data are essential for forensic interpretation in complex cases, such as disputed paternity, distant kinship, and identification following mass disasters. The integration of mutation rate data into statistical models enhances the reliability of likelihood ratios (LRs), improves the analysis of mixed samples, and informs the selection of optimal STR loci for future forensic panels [3,6].

## 6. Forensic Applications and Analytical Methodologies

Autosomal STR loci have emerged as the primary markers for forensic DNA profiling due to their exceptional polymorphism, codominant inheritance, and broad genomic distribution [13,14,25]. The analytical process begins with the targeted PCR amplification of specific STR loci, which produces locus-specific allelic patterns that collectively constitute an individual’s unique genetic profile [5]. Because each locus exhibits a multitude of alleles within a population, the combination of alleles across a panel of multiple loci supports near-unique profiles for every individual, with the notable exception of monozygotic twins.

The implementation of automated CE revolutionized STR analysis by enabling high-throughput, reproducible separation and sizing of PCR products [41,44]. Modern multiplex PCR systems have advanced significantly, with the capacity to simultaneously amplify between 15 and 27 or more loci. This multiplexing improves laboratory efficiency, reduces the consumption of often-limited DNA templates, and drastically enhances the power of discrimination [3,23,45]. Furthermore, STR analysis is remarkably sensitive; it is capable of generating complete genetic profiles from trace amounts of biological material, including hair, urine, and contact “touch” DNA samples from various surfaces [1,14,26].

Accurate allele designation is predicated on the use of allelic ladders, which serve as standardized reference scales for comparison [4,46]. By comparing the migration time or sequence of each PCR product against these ladders, scientists assign correct allele numbers, a step that is essential for maintaining reproducibility and inter-laboratory consistency. These designations are then compared against population-specific allele frequencies to calculate match probabilities and LRs, which form the statistical backbone of forensic evidence evaluation.

STR polymorphisms typically exhibit high heterozygosity rates, ranging from 70% to 90%, reflecting extensive allelic diversity at each locus [11,47]. This inherent variability allows forensic practitioners to differentiate between virtually all unrelated individuals. When multiple loci are analyzed, match probabilities reach extremely low levels; for instance, 20-locus multiplex systems can achieve random match probabilities as low as 10^−24^, ensuring virtually unique identification [23].

The maintenance of population-specific databases is vital for accurate STR interpretation. These repositories include allele frequencies across diverse ethnic and geographic groups, providing the statistical robustness necessary for match probability calculations [4,11]. The integration of sequence-level information through MPS further enhances database resolution, facilitating cross-population comparisons and global forensic casework [24].

Beyond individual identification, STR profiling plays a pivotal role in complex kinship testing, including grandparentage, sibling testing, and postmortem paternity verification [6,48]. To support reliable conclusions, locus-specific mutation rates are incorporated into statistical models to account for potential allelic discrepancies. Sophisticated models now consider factors such as allelic dropout, mutation events, and population substructure, thereby maximizing accuracy for legal and investigative purposes. Additionally, STR profiling is applied in clinical settings for chimerism analysis following hematopoietic stem cell transplantation and for zygosity determination in twins [49]. In these contexts, STR data provide quantitative assessments of donor versus recipient cell populations, enabling the monitoring of engraftment and the early detection of graft failure. Monozygotic twins are identified via identical STR profiles, which is critical for assessing organ transplantation compatibility and conducting genetic research.

## 7. Sex Determination in Forensic STR Analysis

One of the earliest and most extensively utilized methodologies for sex determination in forensic practice is the amelogenin assay, which exploits evolutionary sequence divergence between the X and Y chromosome versions of the amelogenin gene [14,50,51]. This gene encodes a structural protein essential for dental enamel formation; a characteristic small deletion on the X chromosome relative to the Y chromosome allows for the distinct differentiation of male (XY) and female (XX) biological samples through PCR amplification [22,52]. Modern multiplex PCR systems routinely co-amplify the amelogenin marker alongside a suite of autosomal STR loci, providing simultaneous sex determination and individual identity verification, even when analyzing highly degraded biological samples [14,50]. Furthermore, the implementation of sequence-based analysis significantly enhances the robustness of sex determination by enabling the detection of rare allelic variants or mutations within primer-binding sites that could otherwise lead to allelic dropout and subsequent gender misclassification [4].

The ability to accurately determine sex is a fundamental requirement not only for general individualization but also for specialized applications such as postmortem paternity testing, prenatal genetic analysis, and disaster victim identification (DVI). STR-based sex determination has been suggested to be exceptionally reliable in large-scale disaster scenarios and complex forensic investigations where biological tissues may be quantitatively limited or subjected to environmental contamination [53].

However, forensic practitioners must consider potential interference from non-human DNA, particularly when dealing with mixed or contaminated samples. While primers designed for the human amelogenin gene can cross-amplify homologous sequences in other mammalian species, specific differences in fragment lengths typically facilitate accurate differentiation [51,54]. To support the highest level of scientific integrity, rigorous laboratory protocols, the consistent use of negative controls, and verification with additional Y-chromosomal markers remain critical for avoiding the misinterpretation of sex in challenging forensic contexts.

Despite its widespread use, amelogenin-based sex determination is not without limitations. Allelic dropout caused by primer-binding mutations, chromosomal aneuploidies, or structural rearrangements may produce discordant or misleading results. Consequently, confirmatory analysis using supplementary Y-chromosomal markers or sequence-based approaches is recommended, particularly in degraded or low-template samples [14,50]. Amelogenin dropout, chromosomal aneuploidies, and structural variants may produce discordant sex results; therefore, supplementary markers are recommended.

## 8. Evaluation of STR Results

The forensic interpretation of STR DNA evidence provides exceptional discriminatory power, facilitating high-resolution individualization. When utilizing standard commercial kits containing approximately 15–27 STR markers, the probability of identity (PID) typically falls below 10^−15^, while the sibling model (PIDsib) generally remains between 10^−4^ and 10^−6^, ensuring robust discrimination even among close biological relatives [23,55,56]. While these measures describe random match probabilities, they do not directly express the degree of evidential support between competing investigative hypotheses. Consequently, the strength of DNA evidence is evaluated using LR, which compares the probability of the observed DNA profile under the assumption that it originates from the individual of interest versus the probability that it derives from an unrelated person in the relevant population. With the current standard of 16–24 STR loci, LR values can reach extremely high orders of magnitude for unrelated individuals while remaining highly informative for comparisons involving close relatives [57].

The transition from traditional CE to MPS has introduced a critical shift from length-based to sequence-based LR estimation. In traditional CE-based analysis, the LR is constrained by length polymorphism, where different alleles with the same number of repeats are treated as identical. In contrast, sequence-based LR accounts for internal polymorphisms, or isoalleles, which significantly increase the observed allelic diversity within a population [58,59]. By uncovering these hidden variants, the frequency of a specific allele in the population database effectively decreases, which mathematically leads to a second increase in the LR. This advantage is particularly pronounced in complex kinship cases or multi-donor mixtures, where the integration of flanking region SNPs into the sequence-based LR framework provides additional independent data points that further refine the statistical weight of the evidence [3].

Reliable LR estimation necessitates the use of population-appropriate allele frequency databases, and probabilistic genotyping (PG) software model is now the common standard for interpreting complex or mixed samples [60,61]. Modern PG tools utilize Bayesian models to account for the increased complexity of MPS data, including sequence-specific stutter patterns and noise, which require more sophisticated parameters than traditional CE-based models. In criminal investigations, these statistical approaches are central to comparing biological traces with suspects or database entries, where the interpretation of multi-donor mixtures remains a challenging task requiring advanced probabilistic modeling [60,62].

However, the widespread implementation of PG software has sparked significant debate regarding the nature of some commercial algorithms, where proprietary source codes limit independent peer review and full judicial transparency. This lack of transparency can hinder the ability of the defense to scrutinize the underlying mathematical assumptions and software performance. To mitigate these concerns, the forensic community increasingly emphasizes the role of open-source software solutions, such as EuroForMix, which allow for complete algorithmic transparency and facilitate independent validation by the scientific community [63]. Ensuring that computational frameworks are not only statistically rigorous but also transparent and accessible is essential for maintaining the legal defensibility and ethical integrity of forensic DNA evidence [60].

Mutations and null alleles represent primary sources of uncertainty in LR estimation, particularly in kinship cases where they can alter results by several orders of magnitude [64]. Modern forensic approaches incorporate specific mutation models to mitigate this risk, while international guidelines emphasize a probabilistic LR-based interpretation that explicitly accounts for allele frequency uncertainty and population structure [58,65,66,67,68]. The transition from subjective manual interpretation to statistically rigorous computational frameworks supports that DNA interpretation remains reproducible and legally defensible.

## 9. MPS and Integrated STR Analysis

MPS transcends the limitations of conventional CE by providing sequence-level resolution of STR loci [15,69]. While CE is restricted to detecting allele length polymorphisms, MPS simultaneously characterizes the specific repeat number, internal sequence variations, flanking region SNPs, and repeat interruptions. These additional layers of genetic information increase the discriminatory power by 3 to 10 times per locus [4,17,26,59]. A critical technical advantage of MPS is the detection of isoalleles [59], which significantly enhances the deconvolution of minor contributors in complex biological mixtures. Despite these advantages, the practical implementation of MPS in forensic laboratories is influenced by economic and temporal factors. While CE remains the gold standard for routine casework due to its cost-effectiveness and rapid turnaround time, MPS is increasingly reserved for complex multi-donor mixtures and highly degraded samples that require enhanced resolution. The technical efficacy of MPS is particularly evident in the analysis of challenging samples. It enables the profiling of highly degraded DNA and low-template DNA amounts, where CE frequently suffers from allelic dropout [15,16]. Sequence polymorphisms expand the effective allele counts from approximately 15–20 per locus in CE systems to 50–200 per locus in MPS workflows [59]. However, the implementation of MPS introduces specific analytical challenges. Stutter artifacts, previously modeled solely by repeat number, are dependent on complex sequence composition, necessitating the development of locus-specific stutter ratio databases [7,58]. Furthermore, bioinformatics pipelines must manage massive data outputs and utilize standardized variant calling to support inter-laboratory reproducibility [25,58].

While MPS platforms offer exceptional sensitivity, often enabling the generation of profiles from low-copy-number templates, this technical capability necessitates cautious interpretation. At ultra-low-template levels, the analysis is increasingly susceptible to stochastic effects, including significant allele imbalance, allelic dropout, and the potential for drop-in events from minor exogenous contaminants. Consequently, the increased risk of stochastic noise requires the implementation of robust bioinformatic filters and stringent validation protocols to ensure that the detected low-level sequence variants represent the true donor genotype rather than sequencing artifacts or background noise [25,67].

The fundamental differences in analytical resolution, sensitivity, and multiplexing capabilities between CE and MPS platforms are summarized in Table 1.

Mixture interpretation within MPS frameworks relies on PG software, such as EuroForMix, STRmix, and TrueAllele. These tools utilize Bayesian LR models to replace subjective peak-thresholding with statistically rigorous modeling of peak heights, stutter, allelic dropout, and population substructure [58,60,62]. While PG implementation supports court admissibility, technical concerns remain regarding the transparency of proprietary algorithms and the estimation of parameters for drop-in events [58,61,70]. Court admissibility criteria vary between jurisdictions and depend on national legal standards, validation documentation, and expert interpretation practices.

The expansion of sequence-based STR catalogs significantly increases the combined match probability, which is essential for the exoneration of innocent individuals [58,59]. To augment individualization, forensic systems increasingly integrate autosomal STRs with supplementary markers. For instance, Y-chromosomal STRs provide male lineage resolution [71], while mitochondrial DNA (mtDNA) sequencing traces maternal lineages [72]. Insertion/deletion (InDel) markers, characterized by small amplicon sizes and minimal stutter, are particularly advantageous for the study of degraded skeletal remains [73,74]. Targeted MPS assays now incorporate SNPs for pigmentation traits and ancestry-informative SNPs, merging identity testing with phenotypic prediction and biogeographical ancestry inference into single, streamlined workflows [75].

## 10. Analysis of Highly Degraded Biological Materials

The molecular genetic analysis of skeletal remains from mass graves and historical sites requires specialized methodologies to overcome extreme DNA degradation and the presence of potent PCR inhibitors, such as humic acids, fulvic acids, and collagen breakdown products [15,16]. To maximize the recovery of ultra-short genomic fragments, success is fundamentally dependent on optimized extraction protocols targeting dense cortical bone and teeth [76,77]. While both CE and MPS are susceptible to inhibition, they are affected at different stages of the workflow. In CE-based workflows, inhibitors typically interfere with polymerase activity during PCR, leading to partial profiles or total amplification failure. Conversely, in MPS workflows, inhibitors can interfere not only with the initial amplification but also with enzymatic steps during library preparation, such as end repair and adapter ligation. Furthermore, residual inhibitors may affect the clustering process or the sequencing-by-synthesis chemistry, potentially leading to unequal coverage across loci or an increase in sequencing noise. Therefore, the implementation of robust purification methods and the use of engineered polymerases are even more critical when transitioning to MPS for highly compromised skeletal samples [15,25,78,79].

Advanced autosomal STR analysis has been the primary tool for resolving high-profile identification cases in Slovenia. At the Konfin I mass grave site, these methods enabled the definitive molecular identification of victims decades postmortem [76]. In cases of extreme degradation where traditional STR amplicons fail, the implementation of identity and phenotypic SNPs has been suggested as essential for achieving statistically significant LRs, as demonstrated in the identification of a Slovenian pre-war elite couple and victims from various Second World War sites [77,80]. The integration of MPS has further increased success rates by providing a dual layer of genetic information through the simultaneous analysis of STRs and SNPs, which is critical for resolving distant kinship or cases with limited reference samples [15,81,82,83,84]. A significant advantage of transitioning to MPS is its ability to blur the distinction between different types of genetic markers. By facilitating ‘all-in-one’ assays, MPS allows for the simultaneous analysis of STRs and supplementary markers, such as SNPs. This integrated approach is particularly transformative for the study of highly degraded skeletal remains, as it maximizes the information recovery from a single library preparation, providing both high-resolution identification and additional lineage or phenotypic data [85]. This high-resolution capability was exemplified in the kinship analysis of 5th- to 6th-century skeletons from the Bled–Pristava archaeological site, where it enabled the reconstruction of complex familial relationships among Romanized indigenous people [86,87]. Although analytical technologies overlap, statistical interpretation frameworks differ substantially between archaeological inference and forensic individual identification. Transferability of likelihood models should therefore be applied cautiously [86].

When autosomal profiles are partial, the synergy between mtDNA sequencing and Y-STR profiling serves as an indispensable tool for lineage tracing [71,72,76]. Furthermore, InDel markers have emerged as a robust alternative due to their short amplicon sizes, facilitating the profiling of highly compromised remains [73,74]. The success of profiling from ancient or forensic skeletal remains is primarily governed by the state of biomolecular preservation, where environmental factors lead to fragments often shorter than 150 base pairs. To circumvent these limitations, forensic practitioners must employ a hierarchical strategy—ranging from mini-STRs to advanced MPS workflows that leverage the recovery of ultra-short fragments [16,81,82,83,84]. The strategic alignment of analytical methodologies with the specific degree of DNA degradation observed in skeletal remains is summarized in Table 2.

Comparable analytical challenges have been documented in international disaster victim identification and post-conflict investigations across multiple regions, demonstrating that methodological success depends primarily on preservation state, extraction strategy, and validation protocols rather than geographic origin alone. Cross-jurisdictional experience confirms that hierarchical analytical strategies combining STRs, SNPs, and lineage markers represent a broadly transferable forensic framework [84,88].

## 11. Stochastic Effects in STR Analysis

Stochastic effects represent one of the most critical analytical challenges in forensic STR typing, particularly when dealing with low-template DNA, degraded biological material, or complex DNA mixtures. These effects arise from random variation in the early cycles of PCR amplification, where limited template molecules result in unequal allele amplification and reduced reproducibility between replicate analyses [84].

The most prominent manifestations of stochastic behavior include allele dropout, allele drop-in, peak imbalance, and increased stutter proportions. Allele dropout occurs when one allele of a heterozygous locus fails to amplify above analytical thresholds, potentially leading to false homozygous genotype interpretation. Conversely, allele drop-in refers to the sporadic detection of non-contributor alleles, often associated with low-level contamination or sporadic amplification artifacts [65].

Stochastic variation is strongly influenced by template quantity, DNA degradation level, inhibitor presence, and amplification efficiency. Highly degraded samples frequently exhibit preferential amplification of shorter fragments, producing locus-specific imbalance known as heterozygote peak imbalance or locus failure [84]. Such phenomena are particularly relevant in skeletal remains and historical investigations, where DNA fragmentation and environmental damage significantly reduce effective template availability [76,77,80].

Analytical thresholds have therefore been introduced to mitigate stochastic interpretation errors. International recommendations emphasize distinguishing between analytical thresholds, stochastic thresholds, and interpretation thresholds, each reflecting different levels of evidential reliability [65,66]. However, threshold values remain laboratory-dependent and must be established through internal validation studies rather than universally applied parameters.

The transition from CE to MPS modifies—but does not eliminate—stochastic effects. Sequencing-based workflows introduce additional sources of variability, including library preparation efficiency, adapter ligation bias, and sequencing depth variation. While increased read counts can improve sensitivity, ultra-low template samples may still produce allele imbalance and sequence-specific dropout patterns [69,83].

PG systems were developed partly to address stochastic uncertainty by modeling peak height variability and dropout probabilities using statistical frameworks. Continuous models implemented in software such as EuroForMix and TrueAllele incorporate stochastic parameters into likelihood ratio calculations, thereby improving the interpretation of complex mixtures compared with binary threshold-based approaches [63,68].

Importantly, stochastic effects should not be interpreted solely as technical limitations but as predictable statistical phenomena that can be quantified through validation and appropriate probabilistic modelling. Understanding stochastic behavior is therefore essential for avoiding overinterpretation of ultra-low DNA evidence and preventing false certainty in forensic conclusions.

## 12. Validation of STR Typing Methods and Emerging Technologies

Method validation constitutes a fundamental requirement for the admissibility and scientific reliability of forensic DNA evidence. Validation supports that analytical procedures perform consistently under defined laboratory conditions and that interpretation parameters are supported by empirical data. International guidelines issued by the DNA Commission of the International Society for Forensic Genetics (ISFG) emphasize that validation must precede routine implementation of any new forensic genetic methodology [65,66].

Developmental validation evaluates the overall performance characteristics of a method, including sensitivity, specificity, reproducibility, mixture performance, and resistance to inhibitors. Internal validation subsequently confirms performance within individual laboratory environments, accounting for instrumentation, operator variability, and workflow-specific parameters. These processes are particularly critical when implementing new STR kits or transitioning from CE-based typing to MPS platforms [69,81].

Sequencing-based STR analysis introduces additional validation dimensions beyond traditional fragment-length analysis. Laboratories must evaluate sequence concordance, read depth thresholds, strand balance, bioinformatic filtering parameters, and isoallele detection reliability [67,79]. Because MPS workflows include multiple analytical stages—PCR amplification, library preparation, sequencing chemistry, and bioinformatic processing—validation must address each step independently.

Validation studies performed on degraded and challenging samples demonstrate that performance metrics may differ substantially from those observed under optimal laboratory conditions. Studies evaluating MPS-STR kits on compromised biological material report improved recovery of genetic information but also increased sensitivity to stochastic variation and analytical noise [81,83]. Consequently, validation datasets must include realistic forensic scenarios rather than exclusively high-quality reference samples.

An additional validation challenge arises from PG systems, which require verification not only of laboratory performance but also of statistical modeling assumptions. Software transparency, parameter testing, and reproducibility assessments are increasingly emphasized to support judicial acceptance of probabilistic interpretations [63,68].

Importantly, validation should be viewed as a continuous process rather than a single pre-implementation step. As sequencing technologies evolve and new analytical markers are introduced, laboratories must periodically reassess performance metrics, population databases, and interpretation frameworks. This iterative validation approach is essential for maintaining scientific robustness while enabling a responsible adoption of emerging forensic genomic technologies.

Ultimately, standardized validation practices provide the critical bridge between technological innovation and courtroom reliability, ensuring that advances in forensic genetics translate into reproducible and legally defensible evidence.

## 13. Discussion

Forensic genetics has evolved dramatically since the introduction of STR polymorphisms, which now provide the foundational data for kinship verification, individual identification, and zygosity determination [1,3,13,50]. The high heterozygosity and low mutation rates of carefully selected loci support that parentage and sibling testing remain highly reliable [24,41,47,48]. In paternity testing, the application of established protocols, such as the “triple exclusion rule”, remains a critical safeguard against false exclusions caused by rare germline mutations or silent alleles [6,39,48]. Modern multiplex panels, now incorporating up to 27 loci, have significantly elevated the accuracy of these assessments, particularly when analyzing postmortem tissues or cases involving close relatives as potential contributors [3,23,24,45].

The role of STR analysis as the central pillar of individual identification is most profoundly tested in DVI and the analysis of skeletal remains from mass graves [88]. The success of identifying victims from Second World War sites and Slovenian mass graves, such as Konfin I, underscores the necessity of a multifaceted genetic approach [76,77]. By integrating mini-STRs, autosomal SNPs, Y-chromosomal SNPs, and mtDNA, forensic practitioners can now generate informative profiles from templates previously considered “exhausted” due to extreme fragmentation [16,81,82,83,84]. This synergy was historically validated in the Romanov family case and continues to be refined in modern investigations of missing persons and historical skeletal remains [86,89].

Clinical applications further illustrate the versatility of autosomal STRs. In hematopoietic stem cell transplantation, the ability to quantify donor-to-recipient cell ratios (chimerism analysis) provides an essential diagnostic window for early graft failure [49,90] or the differentiation between monozygotic and dizygotic twins [32,49]. However, the increasing sensitivity of these technologies necessitates enhanced vigilance regarding DNA contamination and ethical governance. Stringent laboratory controls are no longer optional but foundational to the integrity of the evidence [32,49]. While MPS offers unprecedented sensitivity for trace DNA, it simultaneously increases the risk of ‘false certainty’ through the detection of stochastic drop-ins. Practitioners must exercise caution, as high-resolution sequence data may inadvertently imbue background contamination with a false sense of evidentiary relevance. At ultra-low quantities, the distinction between a true contributor and a secondary transfer event becomes increasingly precarious [82].

As we move toward sequence-based characterization, the “neutrality” of STR markers remains a key ethical safeguard, ensuring that identity verification does not inadvertently reveal sensitive medical or phenotypic data [13,23]. Nevertheless, the expansion of national databases and the shift toward MPS require robust legal frameworks to prevent unauthorized data use and ensure that the societal benefits of DNA profiling do not compromise individual privacy [3,17]. Population databases remain unevenly distributed geographically, potentially introducing representation bias in isoallele frequency estimation [91]. Beyond technical requirements, laboratories must ensure that the retention of raw sequencing data complies with stringent regulations, balancing evidentiary necessity with the right to data minimization.

The integration of MPS and PG represents the current state-of-the-art, shifting the focus toward total sequence resolution and allowing for the statistical modeling of complex multi-donor mixtures within a Bayesian framework [60,62]. This advancement necessitates rigorous standardization of bioinformatic pipelines to prevent laboratory-specific biases. Maintaining proportionality between scientific capability and ethical responsibility remains essential for sustaining public trust in forensic genetics.

The integration of ancestry-informative and phenotype-predictive markers into forensic workflows introduces ethical and societal considerations beyond traditional identity testing. While such markers may provide investigative intelligence, they also carry risks of misinterpretation, stigmatization, or investigative bias if probabilistic predictions are presented as deterministic conclusions.

Ethical implementation, therefore, requires transparent reporting, clear communication of uncertainty, and jurisdiction-specific legal safeguards. Studies have shown that ancestry inference can generate false investigative leads when population structure and statistical limitations are not sufficiently accounted for [92]. Maintaining proportionality between scientific capability and ethical responsibility remains essential for sustaining public trust in forensic genetics.

## 14. Limitations of This Review

This review synthesizes current developments in autosomal STR analysis and emerging sequencing technologies in forensic genetics; however, several limitations should be acknowledged. First, although a structured literature search strategy was applied, the review remains narrative in nature and therefore does not represent a fully systematic meta-analysis. Differences in study design, laboratory validation protocols, population sampling strategies, and reporting standards limit direct quantitative comparisons across studies.

Second, many cited performance metrics originate from laboratory-specific validation studies conducted under controlled conditions, which may not fully reflect the variability encountered in routine forensic casework. Consequently, analytical thresholds, stochastic effects, and interpretation frameworks should be understood as context-dependent rather than universally transferable.

Third, the geographical representation of casework examples reflects areas with extensive published forensic documentation, including regional expertise, which may introduce a partial regional emphasis despite efforts to include international comparative literature.

Finally, rapid technological development—particularly in MPS, PG, and bioinformatic pipelines—means that some recommendations described here represent evolving best practices rather than finalized global standards.

These limitations highlight the need for continued validation, harmonized reporting standards, and expanded population datasets before universal implementation of emerging methodologies can be achieved.

## 15. Future Research Priorities

Future research priorities in forensic STR analysis center on establishing standardized validation frameworks for MPS-based typing, including inter-laboratory reproducibility studies, as well as improving the characterization of stochastic effects at ultra-low DNA quantities across sequencing platforms. Further development is needed in expanding population reference databases, especially for isoalleles and underrepresented groups, and in defining integration standards for PG and sequencing data, accompanied by transparent reporting guidelines. Additional priorities include conducting cost–benefit and turnaround-time evaluations comparing CE and MPS workflows in operational laboratories, developing ethical and legal frameworks for forensic DNA phenotyping and ancestry inference, and strengthening contamination detection and secondary transfer modelling with robust empirical datasets. Addressing these areas will be crucial for translating technological innovation into reliable and globally harmonized forensic practice.

## 16. Conclusions

Autosomal STR markers remain the globally validated foundation of forensic human identification due to their high discrimination power, standardized nomenclature, extensive population databases, and well-established statistical interpretation frameworks [65,66,67,72]. CE-based STR typing continues to represent the reference methodology in routine forensic practice, supported by decades of developmental and internal validation studies as well as international forensic genetics recommendations [65,66].

At the same time, accumulating evidence demonstrates that sequence-based STR analysis using MPS, together with PG approaches, substantially enhances the interpretation of complex DNA mixtures and degraded samples by enabling sequence-level variation detection and improved statistical modelling [63,68,69,79,81,83]. However, analytical performance and implementation outcomes remain dependent on laboratory-specific validation procedures, bioinformatic workflows, infrastructure availability, and jurisdictional legal frameworks. Current evidence therefore supports an expanding and complementary role for sequencing technologies rather than a universal replacement of established CE-based methodologies [66,69].

Ongoing developments in forensic genetics are expected to focus on expansion of sequence-based population databases, harmonization of analytical and stochastic thresholds, continued validation of sequencing workflows, ethical governance of predictive and ancestry-associated markers, and development of cost-efficient integrated genomic pipelines suitable for routine casework [66,68,79,81,93]. Consequently, the future of forensic genetics is best understood as a progressive integration of validated innovations into established analytical frameworks, guided by empirical evidence, ethical safeguards, and operational feasibility.

## Figures and Tables

**Table 1:** Technical and economic comparison between CE and MPS. Performance parameters are influenced by laboratory validation protocols, sample condition, and analytical workflow. Economic data represents a source-based approximation.

Feature	CE	MPS
**Detection Basis**	Fragment length (amplicon size in bp)	Nucleotide sequence of motifs and flanking regions
**Allelic Resolution**	Low (cannot distinguish isoalleles)	High (detects isoalleles and flanking SNPs)
**Allelic Variation**	Based on repeat number only	Based on the repeat number and sequence variation
**Discriminatory Power**	Standard (e.g., 15–20 alleles/locus)	Enhanced (e.g., 50–200+ alleles/locus)
**Mixture Interpretation**	Limited by peak overlaps and stutter	High resolution via unique sequence reads
**Sensitivity**	High (routine casework)	Extremely high (effective for low-copy-number DNA)
**Multiplex Capacity**	Limited by fluorescent dye channels (~30 loci)	Massive (hundreds of loci in a single run)
**Setup cost**	lower	higher
**Throughput**	moderate	high
**Resolution**	length-based	sequence-based
**Turnaround**	faster single sample	efficient batch

**Table 2:** Strategic selection of analytical methodologies based on the degree of DNA degradation in forensic and archaeological skeletal remains. Values represent an orientative framework derived from multiple studies and should not be interpreted as universal analytical thresholds.

Degradation Level	DNA Fragment Size (bp)	Recommended Analytical Methodology	Statistical Significance (LR)
**Low**	>400 bp	standard commercial autosomal STR kits	Extremely High
**Moderate**	150–250 bp	mini-STRs	High
**High**	50–150 bp	SNPs integrated with MPS platforms	Moderate to High
**Extreme**	<50 bp	mtDNA (Control Region)	Lineage-specific

## Data Availability

No new experimental data were generated in this study. A curated body of reviewed literature forms the evidentiary basis of this narrative synthesis.

## Supplementary Materials

The following supporting information can be downloaded at: https://www.mdpi.com/article/10.3390/genes17030285/s1, Table S1, Evidence Level and Risk of Bias Assessment of Reviewed Studies.
