# Supplementary material for: Autosomal STR Markers for Forensic Genetics: Applications, Challenges, and Future Directions
Source: Genes (Basel). 2026 Feb 27;17(3):285. doi: 10.3390/genes17030285 (PMC13025442; doi:10.3390/genes17030285)
Supplement: Supplementary file 1 [file genes-17-00285-s001.zip › genes-4152627-supplementary.pdf]

Table S1. Evidence Level and Risk of Bias Assessment of Reviewed Studies

Study Type	Typical Examples in Review	Evidence Level	Potential Bias Sources
Guidelines / Standards	ISFG recommendations, DNA Commission reports	High	Consensus-driven, not empirical testing
Validation Studies	STR kit validation, MPS performance studies	High–Moderate	Laboratory-specific conditions
Population Studies	Allele frequency databases	Moderate	Population sampling bias
Casework Reports	Historical/skeletal identifications	Moderate–Low	Case-specific interpretation
Methodological Reviews	Technology comparisons CE vs MPS	Moderate	Narrative synthesis limitations
